# An epidemiological study of extracted mandibular premolars from adolescent patients in Damascus using two classification system analyzed with CBCT and digital periapical radiographs

**DOI:** 10.1186/s12903-025-06838-x

**Published:** 2025-09-26

**Authors:** Yasser Alsayed Tolibah, Mohammed N. Al-Shiekh, Mohammad Tamer Abbara, Marwan Alhaji, Osama Aljabban, Nada Bshara

**Affiliations:** 1 Department of pediatric dentistry, Damascus University, P.O. Box 3062, Damascus,, Syria; 2 Department of Endodontics, Damascus University, P.O. Box 3062 Dr, Damascus, Syria

**Keywords:** Damascus population, Dental digital radiography, Dental pulp, Epidemiology, Mandibular premolars

## Abstract

**Objective:**

This study aimed to assess the root number, length, and root canal morphology of extracted mandibular premolars (MPs) from adolescents in Damascus using two classification systems: Vertucci’s classification and Ahmed’s classification. This was conducted through radiographic examination utilizing Cone Beam Computed Tomography (CBCT) and digital dental radiography analysis. Additionally, the study sought to investigate the correlation between these variables and gender within the sample.

**Materials and methods:**

Immediately after extraction, the root number and maximum length were recorded. Each MP was then radiographed in buccolingual and mesiodistal directions using digital periapical radiographs. Subsequently, MPs were scanned using a CBCT device (200 μm voxel size) and the root canal morphology was classified based on Vertucci’s classification and Ahmad’s classification based on both radiographs. Moreover, the presence of C-shaped canals was investigated and classified according to the Fan et al. classification. Finally, the previous findings were correlated with the patient’s gender, and statistical analysis was performed using the Chi-Square test and T-test in the SPSS program. The confidence interval used for the Chi-square test was 95%. Moreover, the sensitivity, accuracy, specificity, and overall agreement tests was performed to compare digital sensor radiographs with the gold standard CBCT radiographs.

**Results:**

The sample included 255 first MPs and 163 s MPs. No significant differences were found between males and females regarding the frequency of one, two, or three roots in the first and second MPs (*p* = 0.931 and *p* = 0.010, respectively). Most MPs had a single root. The mean length of the first MP was 21.9 mm, and the second MP was 21 mm, with males generally having longer MPs than females (*p* < 0.001). There were no significant gender differences in the distribution of first and second MPs according to Vertucci and Ahmed classifications (*p* > 0.05). Type I (^1^TN^1^) was the most common root canal morphology, followed by Type V (^1^TN^1–2^). Low occurrence of C-shape canals was observed in first and second MPs (4.31% and 9.82%, respectively). The sensitivity, specificity, accuracy, and overall agreement between the two radiographic assessment techniques of both MPs were highly comparable.

**Conclusion:**

It can be concluded that variations in root number and canal classifications are evident in MPs. Although gender does not influence the root number or canal morphology, males tend to have longer MPs. Moreover, it is noteworthy that digital sensors performed better in detecting small accessory canals in the apical third.

## Introduction

The morphology of dental roots is intricate and diverse, making a thorough understanding of the pulp anatomy and structure essential for successful endodontic treatment [1]. Failure to detect root canals may lead to endodontic treatment failure due to remaining bacteria in untreated canals [2]. The anatomical complexities of teeth are associated with personal factors such as age, gender, and genetic factors related to race and ethnicity. Therefore, identifying variations among different populations is essential [3–5].

The first and second mandibular premolars (MPs) typically have a single root with one canal. However, the number of roots and the canal anatomy of the first and second MPs can be complex and variable. The literature indicates that a small percentage of MPs exhibit multiple roots or canals, posing potential challenges for endodontic treatment [6, 7]. These anatomical variations can complicate the procedure [8].

Several methods are available to assess canal morphology, such as two-dimensional periapical radiography [9], cone-beam computed tomography (CBCT) [10], micro-computed tomography (micro-CT) [11], dye injection and clearing techniques [7, 12], and photographic imaging with 24x magnification of methylene blue-stained cross-Sect. [13].

It is important to note that each of these methods has its limitations. Traditional radiography is affected by superimposition as a two-dimensional representation of a three-dimensional structure [14]. Factors such as increased image noise, X-ray beam divergence, technical errors related to the surface sensors in CBCT devices (often in small anatomical details), deformation and superimposition of dental features, scattering, extinction artifacts, beam hardening artifacts, exponential edge gradient effects, aliasing artifacts, ring artifacts, and motion artifacts may limit the diagnostic accuracy of in vivo CBCT investigations [15–19]. Moreover, a minimum voxel resolution of 300 microns is required in CBCT devices to capture images for assessing the shape of hard tissues [20]. Additionally, the ionizing radiation in CBCT poses potential risks to the patient [21]. The clearing technique may cause deformation of the tooth structures due to demineralization, and the injected dye may not fully reach the accessory canals. Canal obstruction caused by narrowing and calcification also limits the penetration of the injected dye particles [21]. Finally, methods requiring longitudinal or cross-sectional preparation of teeth for assessment may disturb the pulp shape and surrounding structures or remove small accessory canals during the preparation process [21, 22]. The limitations of previous evaluation methods highlight the need for combining multiple techniques to achieve a more precise and reliable assessment of canal morphology.

Recent studies have evaluated variations in root and root canal morphology of the first and second MPs in different populations, including Italian [23], Chinese [11], Caucasian [24], Saudi [25, 26], German [27], Iranian [28], black South African [29], Turkish [30], Spanish [31], Thai [32], Kuwaiti [33], and northern Syrian populations [7]. Recent studies have also described the morphology and symmetry of the maxillary premolars, maxillary first molars, and maxillary second molars in samples from Damascus residents [10, 34, 35]. However, no recent studies have yet detailed this information in MPs in the Damascus population. It was also noted that the comparison between digital periapical radiography and CBCT remains limited in its evaluation of root canal morphology, as previous comparisons have primarily focused on assessing proximal dental caries [36], alveolar bone levels [37], and diagnosing furcation perforations [38]. Therefore, this study aimed to evaluate the root number, length, and radiographic assessment of the root canal morphology of extracted first and second mandibular premolars (MPs) using CBCT and digital sensor radiography as a primary objective in a sample of adolescents from Damascus. The evaluation was conducted using two classification systems: Vertucci’s classification [39] and the new classification system by Ahmed et al. [40]. The primary null hypothesis proposes that gender does not influence the number of roots, their length, or the canal morphology of MPs. As a secondar objective, this study aimed to assess the sensitivity, specificity, accuracy, and agreement of the digital sensor radiographs taken in both buccolingual and mesiodistal directions compared to gold standard (CBCT radiographs [41]). The secondary null hypothesis proposes that the sensitivity, specificity, accuracy, and agreement are comparable between the two imaging techniques for mandibular premolars in the current sample.

## Materials and methods

### Study design, ethics approval and consent to participate

This cross-sectional epidemiological study was conducted between May 15, 2023, and March 31, 2025, in the Paediatric Dentistry Department at the Faculty of Dentistry, Damascus University. The study design and informed consent process were conducted following the ethical principles of the Helsinki Declaration. The research was approved by the Faculty of Dentistry’s local Ethics Committee (UDDS-361-13032023/SRC-2654). This study was registered in the ISTRCN database (Trial ID: ISRCTN93024803) on 18/03/2025. The extracted premolars were collected as part of an in-vitro study investigating microleakage and fracture resistance in teeth treated with different endodontic treatment procedures. Informed consent was obtained from all subjects and their parents involved in the study, stating that the extracted premolar would be used in an epidemiological study.

## Sample size calculation

Based on previous studies [7, 31, 42] that considered the gender influence on variations in root canal morphology, the sample size for the current study was determined using G* Power 3.1.9.4 (Heinrich Heine University, Düsseldorf, Germany). The Chi-square analysis concluded that the minimum required sample size was 160 samples in each group, corresponding to an effect size (w) of 0.3 with a power of 85% at a significance level of 0.05.

## Sample process

The sample consisted of recently extracted first and second MPs during orthodontic treatment of adolescents aged 12–15 years from Damascus at the Paediatric Dentistry, Orthodontics, and Oral and Maxillofacial Surgery Departments, Faculty of Dentistry, Damascus University. Premolars that had undergone endodontic treatment, or had internal/external resorption, deformities, open apices, or fractures during extraction were excluded. Therefore, 255 first and 163 s MPs met the inclusion criteria. Each extracted MP was assigned a number, and the patient’s gender was recorded to accompany each premolar’s data. Soft tissues, bone fragments, and calculus were removed through scaling and polishing. At this stage, the number of roots and the maximum length of premolars were determined in millimetres with a digital caliper (WEN Digital Caliper, Performance Tool - Wilmar LLC, Kent, Washington, USA). Subsequently, two periapical radiographs were taken for each extracted MP one in the buccolingual direction and one in the mesiodistal direction. This was done using a portable radiographic unit (PRU) (HyperLight, Eighteeth; Changzhou Sifary Medical Technology Co., Ltd., Changzhou City, Jiangsu Province, China) with the following settings: exposure time of 0.08 s, 65 kVp, and 2.5 mA. The PRU was positioned perpendicular to the tooth’s long axis and the surface of the digital sensor (EzSensor HD; Vatech, Gyeonggi-do, Korea) in both radiographs using the parallel technique. The methodology was similar to that described by Faraj et al. [42].

Subsequently, premolars were fixed on 5 boards (about 70 MPs in each board) and scanned with a CBCT device (Computed Tomography X-ray System PHT-6500, Gyeonggi-do, Korea). The imaging parameters included a field of view of 120 × 90 mm, an intensity of 15 mA, a voltage of 120 kV, a voxel size of 0.2 mm (standard resolution mode), and an exposure time of 24 s. The acquired image data were processed using EZDent-i software (Gyeonggi-do, Korea). The investigators conducted image evaluation and data analysis by examining the images in multiple planes, assessing roots and canals at different levels (axial, coronal, sagittal, and oblique). The evaluation was performed from the pulp chamber to the root apex and from mesial to distal, utilizing various imaging adjustments such as brightness, density, contrast, inversion, and sharpness filters. A slice thickness of 0.25 mm was applied to ensure optimal visibility.

## Outcomes measurement

Each MP was analysed and assessed using a digital sensor in both the buccolingual and mesiodistal directions, as well as CBCT sections, by the evaluators to determine a single, accurate classification for each MP. At the same time, the individual assessment from each method is retained to compare sensitivity, specificity, accuracy, and agreement between the two techniques (digital sensors compared to CBCT, which is considered the gold standard). Initially, Vertucci’s classification [39] was used to assess the canal morphology: Any additional types were recorded. As a next step, the modern numbering system by Ahmed et al. [40] was utilized. Finally, the presence of C-shaped root canals was assessed based on the criteria described by Fan et al. [43].

Two specialists with PhD degree from the Faculty of Dentistry (one from the Pediatric Dentistry Department and the other from the Endodontics Department) evaluated the CBCT and digital sensor radiographs together. They re-examined the images after 15 days to ensure the accuracy of the recorded results. The readings of the assessors were entirely consistent, and there was no difference between them. Subsequently, 10% of the premolars were randomly selected, and their radiographs were reviewed by a third specialist with PhD degree from the Endodontics Department to confirm the reliability of the previous evaluators’ results (using Cohen’s kappa test; K = 0.95 *p* < 0.001).

### Statistical analysis

Data were analyzed using SPSS (Version 20, IBM SPSS Inc., Chicago, IL, USA). The Chi-square test was used to compare the distribution of the first and second MPs according to the number of roots and Vertucci classification among male and female participants in the sample. The confidence interval used for the Chi-square test was 95%. Moreover, the sensitivity, accuracy, specificity, and overall agreement tests was performed to compare digital sensor radiographs with the gold standard CBCT radiographs.

## Results

**Fig. 1:**
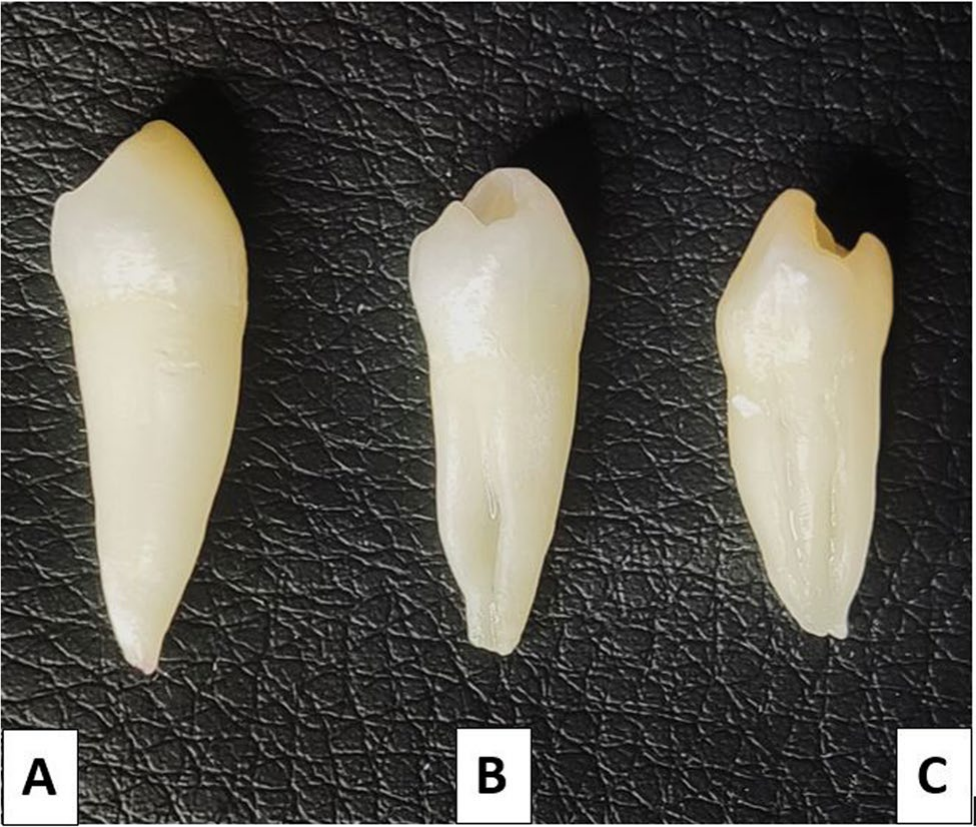
Examples of mandibular first premolars: **A**: with one root, **B**: with two roots, and **C**: with three roots

**Fig. 2:**
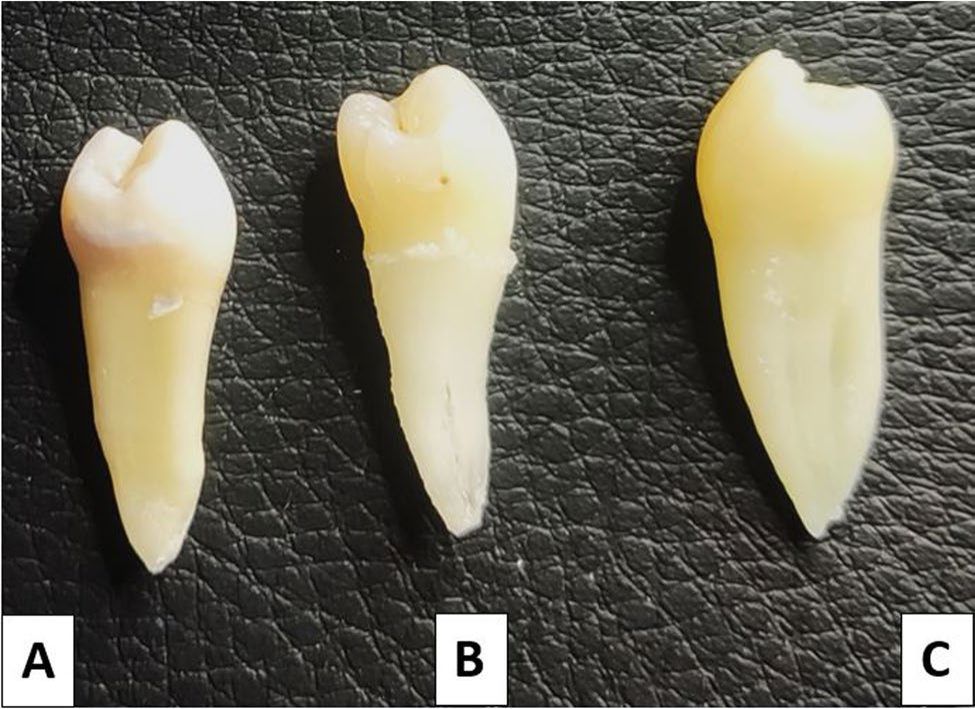
Examples of mandibular second premolars: **A**: with one root, **B**: with two roots and C: with three roots

The sample included 255 first MPs (100 from males and 155 from females) and 163 s MPs (82 from males and 81 from females). Table 1 illustrates the distribution of first and second MPs according to the number of roots among the male and female participants in the sample. The Chi-square test showed no statistically significant differences in the occurrence of one, two, or three roots between males and females in the first and second MPs (*p* = 0.931, *p* = 0.919; respectively). Most MPs had a single root observed in 85.1% of the first and 91.26% of the second. Moreover, two roots were found in 12.5% of first and 8.12% of second MPs. Notably, three root occurrences were rare, found in only 2.3% of first MPs and 0.62% of second MPs (Figs. 1 and 2).

**Table 1:** Distribution of the first and second MPs by the number of roots between males and females in the sample and the results of the Chi-square test

Premolar		First Premolar			Second Premolar		
Gender		Male	Female	Total	Male	Female	Total
Roots Number	One Root	86	131	217 85.1%	76	73	149 91.26%
	Two Roots	12	20	32 12.5%	6	7	13 8.12%
	Three Roots	2	4	6 2.3%	0	1	1 0.62%
Chi-Square Test Result	Chi-Square Value	0.142			0.164		
	*p*-Value	0.931			0.919		

Table 2 presents the mean lengths of the first and second MPs for males and females. The mean length of the first MPs in males was 22.9 mm, and in females was 20.35 mm, with statistically significant differences indicating that males tend to have longer first MPs (*p* < 0.001). Similarly, for the second MP, the mean length in males was 21.3 mm, and in females was 19.75 mm (*p* < 0.001). The overall mean length of the first MPs was 21.9 mm, slightly longer than that of the second (21 mm).

**Table 2:** Lengths of the first and second mandibular premolars in mm among males and females in the sample

Gender	Number	First Premolar Length				Number	Second Premolar Length			
		Mean ± Standard Deviation	Range	T-value	*p-value		Mean ± Standard Deviation	Range	T-value	*p-value
Male	100	22.9 ± 2.2	18–26	9.39	< 0.001^	82	21.3 ± 1.94	18–24	4.59	< 0.001^
Female	155	20.35 ± 1.8	17–24			81	19.75 ± 1.91	17–23		
Total	255	21.9 ± 1.9	17–26			163	21 ± 1.93	17–24		

* T-test

^ Significant difference

**Fig. 3:**
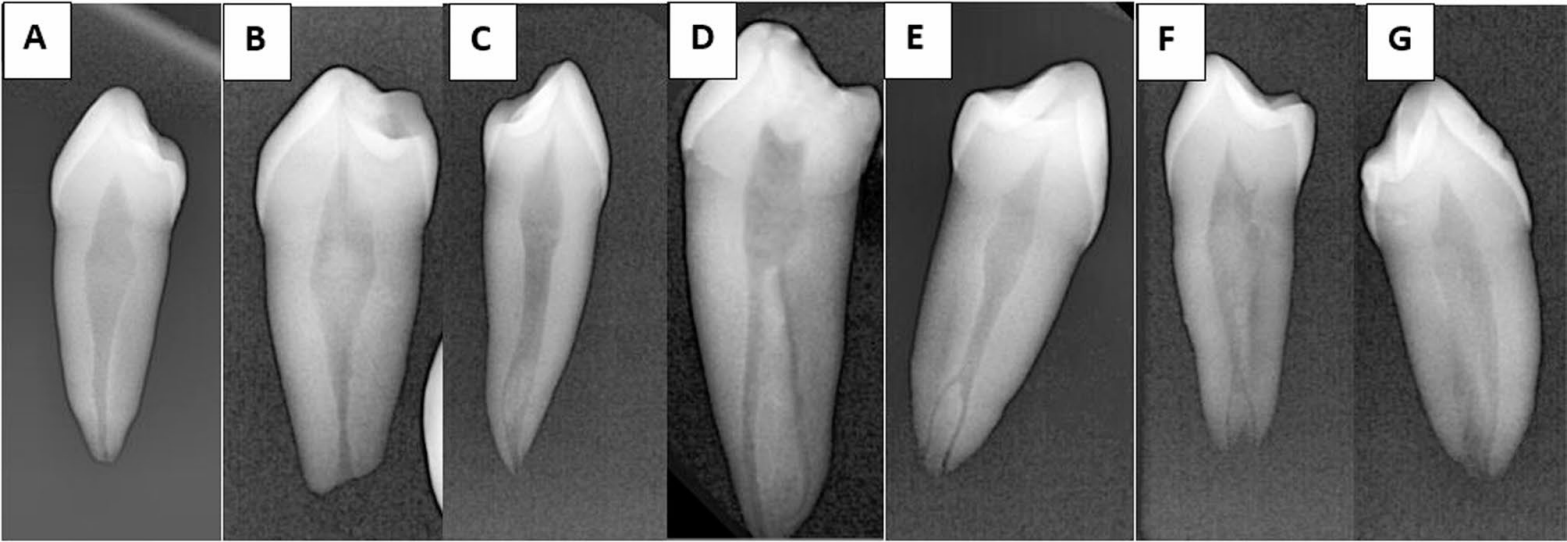
Examples of digital periapical radiographs of mandibular premolars: **A**: with Vertucci Type 1, **B**: with Vertucci Type II, **C**: with Vertucci Type III, **D**: with Vertucci Type IV, **E**: with Vertucci Type V, **F**: with Vertucci Type VI, and **G**: with ^2^TN^1^B^1^L^1^ according to Ahmed Classification

Table 3 illustrates the distribution of first and second MPs according to Vertucci’s canal morphology classification among the males and females in the sample (Fig. 3). The Chi-square test showed no significant differences in the frequency of Vertucci’s classification types between males and females in the single-rooted first and second MPs (*p* = 0.751, *p* = 0.919, respectively) and. The first type had the highest frequency in the first and second MPs (61.75% and 79.45%, respectively), followed by the fifth type (17.51% and 8.72%, respectively), the fourth type (13.82% and 8.21%, respectively, the second type (3.7% and 3.42%, respectively), the sixth type (0.90% and 0.68%, respectively) and the seventh type (0.45% and 0.68%, respectively) The third type was observed only in the first MPs (1.84%). In contrast, this pattern was absent in the second MPs. In contrast, Type VI was observed only in the second MPs (0.68%) and was lacking in the first MPs. Notably, the eighth type was not found in either MP. It is worth noting that a distinct anatomical configuration not included in Vertucci’s classification—specifically, a 3-1-2 pattern—was identified in a single case of second MPs (0.68%).

Regarding MPs with type I Sensitivity and NPV were both 100%, indicating that all cases classified as Type I by CBCT were correctly identified by the digital sensor, and no Type I cases were missed. The PPV was slightly lower at 97.7%, meaning a small number of overcalls may have occurred. The specificity was 96.4%, with an overall accuracy of 98.6% and an agreement rate of 97.6%, highlighting the digital sensor’s reliability for identifying this straightforward canal anatomy. The main issue was the presence of an accessory canal in the apical third in some cases, which altered the classification to Vertucci Type V. This canal was not visible at all in some cases on the CBCT images, while it was clearly identifiable in the digital sensor radiographs. For Type II, sensitivity and NPV were also 100%, while the PPV was 86.6%, suggesting that the digital sensor tended to overestimate the presence of this type in a few cases. Despite this, specificity and accuracy remained high (99.5%), with strong accuracy (99.5%) between the two modalities. In contrast, Type III showed slightly lower sensitivity at 83.3%, suggesting that one case was missed by the digital sensor. However, PPV and specificity were both 100%, and overall accuracy remained high at 99.8%. Types IV, V, and I-I demonstrated near-perfect or perfect diagnostic performance across all tools. Type IV, for instance, had 100% sensitivity, specificity, PPV, NPV, and accuracy. Type V, though slightly lower in sensitivity (90.2%), had a PPV of 100%, indicating no false positives, with excellent accuracy (98.8%). The lowest sensitivity was observed in Type VI at 33.3%, meaning that only one out of three true cases was detected using the digital sensor. However, the PPV was 100%, showing no false positives, and NPV and specificity remained high (99.5% and 100%, respectively). Some canal configurations, such as Type VII, Additional Type ([3–1–2), and Type II in the lingual root of double rooted mandibular MPs, showed low frequency among first and second MPs, which statistically prevented a valid comparison of the sensitivity of the two techniques.

**Table 3:** Distribution of the first and second MPs by vertucci’s Canal morphology classification between males and females in the sample the results of the Chi-square test, sensitivity, positive predictive value, negative predictive value, specificity, accuracy, and agreement of digital sensor images compared to CBCT radiography

Root Numbers	Vertucci Classification	The accurate classification after considering both evaluations.						Digital sensor images compared to CBCT radiography in all mandibular premolars’ radiographs					
		First Premolar			Second Premolar			Sensitivity	Positive Predictive Value	Negative Predictive Value	Specificity	Accuracy	Agreement
		Male	Female	Total	Male	Female	Total						
Single-Rooted Mandibular Premolars	Type I	54	80	134 61.75%	63	53	116 79.45%	100.0% (250/250)	97.7% (250/256)	100.0% (162/162)	96.4% (162/168)	98.6% (412/418)	97.6% (408/418)
	Type II	4	4	8 3.7%	1	4	5 3.42%	100.0% (13/13)	86.6% (13/15)	100.0% (403/403)	99.5% (403/405)	99.5% (416/418)	
	Type III	2	2	4 1.84%	0	0	0 0%	83.3% (5/6)	100.0% (5/5)	99.8% (412/413)	100.0% (412/412)	99.8% (417/418)	
	Type IV	10	20	30 13.82%	5	7	12 8.21%	100.0 (42/42)	100.0% (42/42)	100.0% (376/376)	100.0% (376/376)	100.0% (418/418)	
	Type V	16	22	38 17.51%	6	7	13 8.72%	90.2% (46/51)	100.0% (46/46)	98.7% (367/372)	100.0% (367/367)	98.8% (413/418)	
	Type VI	0	2	2 0.90%	0	1	1 0.68%	33.3% (1/3)	100.0% (1/1)	99.5% (415/417)	100.0% (415/415)	99.5% (416/418)	
	Type VII	0	1	1 0.45	1	0	1 0.68%	N/A	N/A	N/A	N/A	N/A	
	Additional Type (3-1-2)	0	0	0 0%	0	1	1 0.68%	N/A	N/A	N/A	N/A	N/A	
	Chi-Square Value	0.960			0.419			-	-	-	-	-	
	*p*-Value	0.916			0.981			-	-	-	-	-	
Double-Rooted Mandibular Premolars (Buccal Root)	Type I	12	19	31	6	7	13						
	Type II	0	1	1	0	0	0	N/A	N/A	N/A	N/A	N/A	
	Chi-Square Value	0.110			0.010			-	-	-	-	-	
	*p*-Value	0.751			0.919			-	-	-	-	-	
Double-Rooted Mandibular Premolars (Lingual Root)	Type I	12	20	32	6	7	13	100.0% (51/51)	98.1% (51/52)	100.0% (366/366)	99.7% (366/367)	99.8% (417/418)	
	Chi-Square Value	0.125			0.010			-	-	-	-	-	
	*p*-Value	0.724			0.919			-	-	-	-	-	
Triple-Rooted Mandibular Premolars (Mesio-Buccal)	Type I	2	4	6	0	1	1	100.0% (7/7)	100.0% (7/7)	100.0% (411/411)	100.0% (411/411)	100.0% (7/7)	
	Chi-Square Value	0.1778			0.139			-	-	-	-	-	
	*p*-Value	0.673			0.709			-	-	-	-	-	
Triple-Rooted Mandibular Premolars (Distal-Buccal)	Type I	2	4	6	0	1	1	100.0% (7/7)	100.0% (7/7)	100.0% (411/411)	100.0% (411/411)	100.0% (7/7)	
	Chi-Square Value	0.1778			0.139			-	-	-	-	-	
	*p*-Value	0.673			0.709			-	-	-	-	-	
Triple-Rooted Mandibular Premolars (Lingual)	Type I	2	4	6	0	1	1	100.0% (7/7)	100.0% (7/7)	100.0% (411/411)	100.0% (411/411)	100.0% (7/7)	
	Chi-Square Value	0.1778			0.139			-	-	-	-	-	
	*p*-Value	0.673			0.709			-	-	-	-	-	

Table 4 illustrates the distribution of first and second MPs according to Ahmed’s canal morphology classification among the male and female participants in the sample. The majority of first and second MPs were with ^1^ TN ^1^ configuration (52.55% and 71.16%, respectively) and showed high diagnostic performance with a sensitivity of 97.6%, a perfect Positive Predictive Value (PPV) of 100%, and an overall accuracy of 98.6%, indicating that the digital sensor reliably identified this common canal type. The agreement between the digital sensor and CBCT for this configuration was also high at 97.4%. The second most common configuration of first and second MPs, ^1^ TN ^1–2^ (14.9% and 7.97%, respectively), demonstrated perfect sensitivity (100%) but a slightly lower PPV of 90.2%, indicating that while all true cases were detected, some cases identified by the digital sensor may have been overestimated compared to CBCT. However, the overall accuracy remained high at 98.7%. Rare and complex configurations, such as ^1^ TN ^2−1−2^, showed perfect sensitivity and Negative Predictive Value (NPV). Still, the PPV was considerably lower (33.3%), suggesting a tendency of the digital sensor to over-diagnose this rare pattern. Despite this, accuracy remained high (99.5%), and specificity was near perfect (99.5%), showing that misclassifications were limited to only a few cases. Other uncommon types, such as ^2^ TN B ^1^ L ^1^, ^2^ TN ^1^ B ^1^ L ^1^, and ^3^TN MB^1^ DB^1^ L^1^, achieved 100% PPV, NPV, specificity, and accuracy, reflecting excellent agreement with CBCT. For instance, ^3^TN MB ^1^ DB ^1^ L ^1^ demonstrated perfect diagnostic performance across all metrics, confirming the digital sensor’s reliability even in more complex canal structures, when present. Some canal configurations, such as ^1^ TN ^1–2−1–2^, ^1^ TN ^3−1−2^, and ^2^ TN B ^2−1^ L ^1^, showed low frequency among first and second MPs, which statistically prevented a valid comparison of the sensitivity of the two techniques. The statistical analysis indicated that the digital sensor tended to overdiagnose additional apical canals that were not detected in CBCT images. Although this was considered an overdiagnosis from a purely statistical standpoint, in reality, these additional canals were indeed present, suggesting that the digital sensor provided a more accurate reflection of the true canal anatomy in these specific cases. It is worth noting that the classification proposed by Ahmed et al. effectively addressed the challenge of representing both the number of roots and canal morphology in a simplified, accurate, and easily interpretable manner. Figure 3 illustrates some of noted classifications in this sample.

**Table 4:** Distribution of the first and second MPs by ahmed’s Canal morphology classification between males and females in the sample the results of the Chi-square test, sensitivity, positive predictive value, negative predictive value, specificity, accuracy, and agreement of digital sensor images compared to CBCT radiography

Ahmed Classification	The accurate classification after considering both evaluations.						Digital sensor images compared to CBCT radiography in all mandibular premolars’ radiographs					
	First Premolar			Second Premolar			Sensitivity	Positive Predictive Value	Negative Predictive Value	Specificity	Accuracy	Agreement
	Male	Female	Total	Male	Female	Total						
^1^TN^1^	54	80	134 52.55%	63	53	116 71.16%	97.6% (250/256)	100.0% (250/250)	96.43% (162/168)	100.0% (162/162)	98.6% (412/418)	97.4% (407/418)
^1^TN^1–2^	16	22	38 14.9%	6	7	13 7. 97%	100.0% (46/46)	90.19% (46/51)	100.0% (367/367)	98.6% (367/372)	98.7% (413/418)	
^1^TN^1–2−1^	2	2	4 1.57%	0	0	0 0%	100.0% (5/5)	83.3% (5/6)	100.0% (412/412)	99.7% (412/413)	99.8% (417/418)	
^1^TN^1–2−1–2^	0	1	1 0.39%	1	0	1 0.61%	N/A	N/A	N/A	N/A	N/A	
^1^TN^2^	10	20	30 11.75%	5	7	12 7.63%	100.0% (42/42)	100% (42/42)	100.0% (376/376)	100.0% (376/376)	100.0% (418/418)	
^1^TN^2−1^	4	4	8 3.13%	1	4	5 3.01%	86.7% (13/15)	100.0% (13/13)	99.5% (403/405)	100.0% (403/403)	99.5% (416/418)	
^1^TN^2−1−2^	0	2	2 0.78%	0	1	1 1.13%	100.0% (1/1)	33.3% (1/3)	100.0% (415/415)	99.52% (415/417)	99.5% (416/418)	
^1^TN^3−1−2^	0	0	0 0%	0	1	1 0.61%	N/A	N/A	N/A	N/A	N/A	
^2^TN B^1^ L^1^	6	7	13 5.1%	3	4	7 4.3%	95.2% (20/21)	100.0% (20/20)	99.7% (397/398)	100.0% (397/397)	99.8% (417/418)	
^2^TN^1^B^1^L^1^	6	12	18 7.05%	3	3	6 3.68%	100.0% (24/24)	100.0% (24/24)	100.0% (394/394)	100.0% (394/394)	100.0% (418/418)	
^2^TN B^2−1^ L^1^	0	1	1 0.39%	0	0	0 0%	N/A	N/A	N/A	N/A	N/A	
^3^TN MB^1^ DB^1^ L^1^	2	4	6 2.35%	0	1	1 0.61%	100.0% (7/7)	100.0% (7/7)	100.0% (411/411)	100.0% (411/411)	100.0% (418/418)	
Chi-Square Value	0.646			2.831								
*p*-Value	0.958			0.587								

**Fig. 4:**
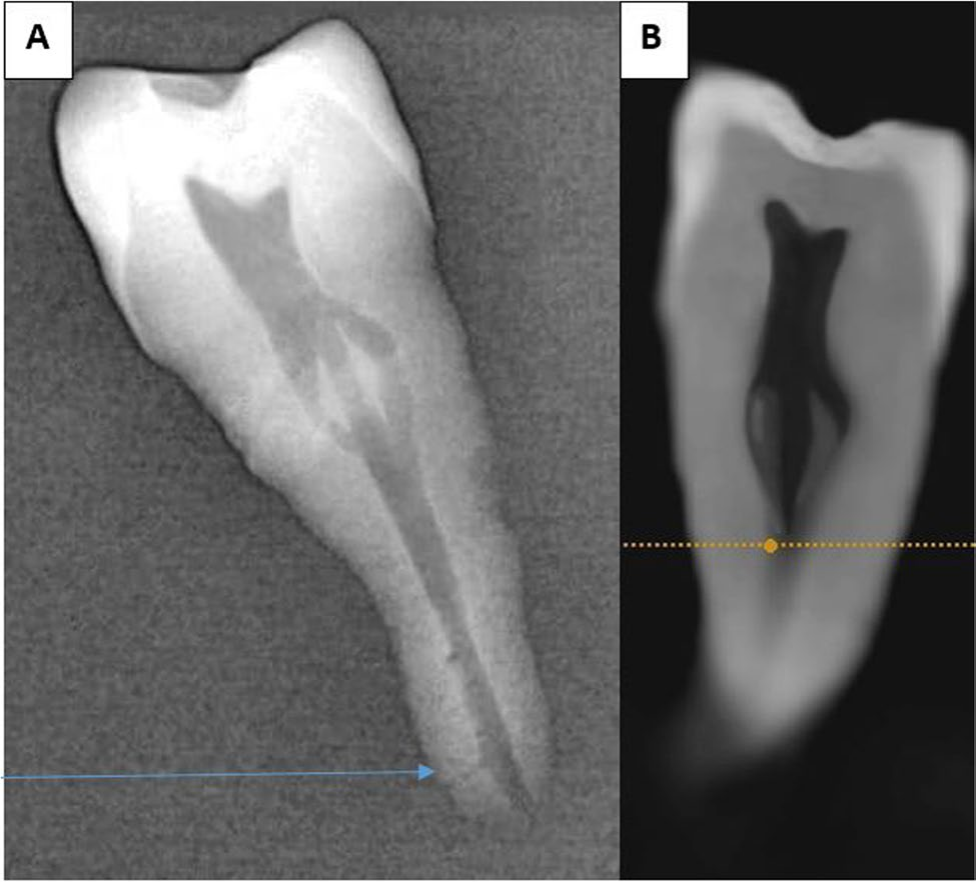
The additional classification observed in a second MP identified as 3-1-2 according to Vertucci’s classification and ^1^TN ^3−1−2^ according to Ahmed’s classification. **A**: captured using digital sensor imaging **B**: one of the CBCT slices. The arrow highlights the small additional canal, which altered the classification and was detected in the digital sensor image but was not identified in the CBCT sections

**Fig. 5:**
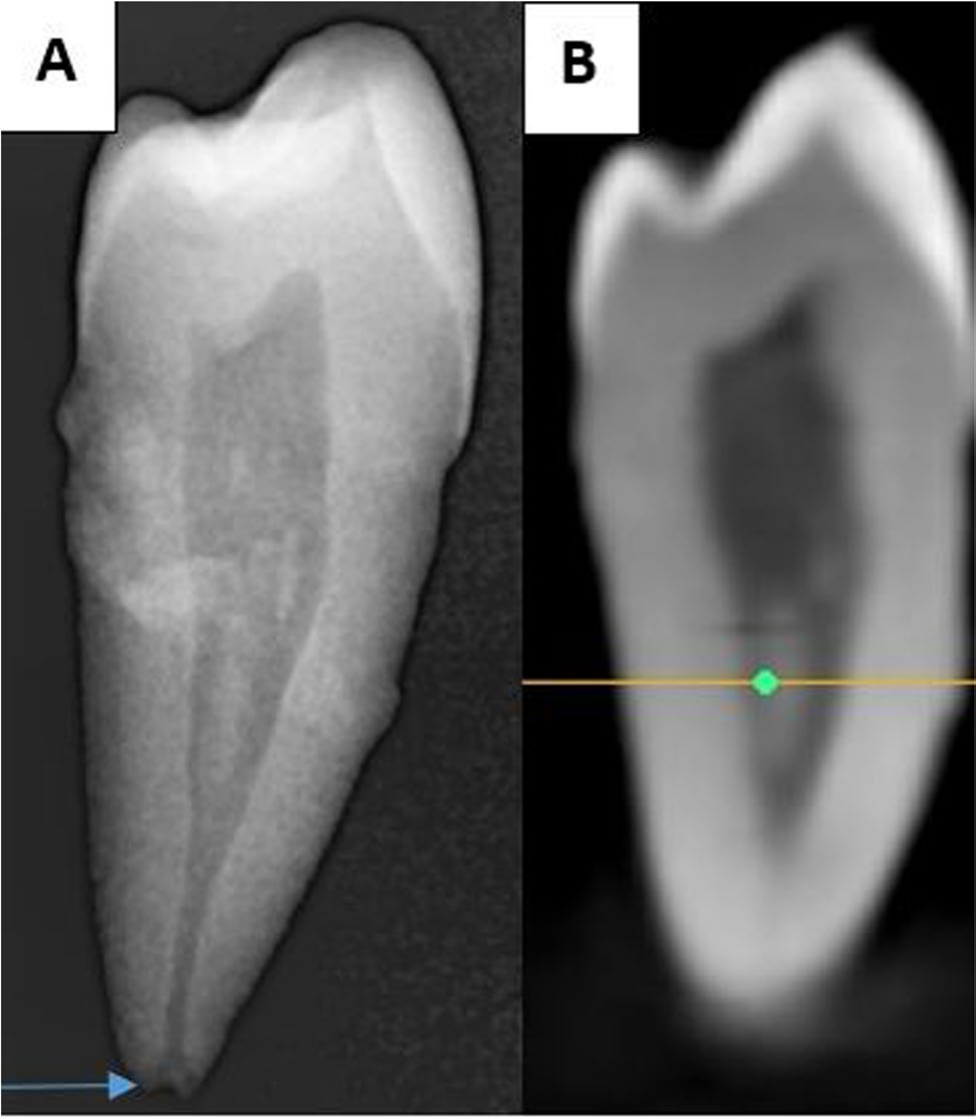
Vertucci class VII or Ahmed ^1^TN^1–2−1–2^. **A**: captured using digital sensor imaging **B**: one of the CBCT slices. The arrow highlights the small additional canal, which altered the classification and was detected in the digital sensor image but was not identified in the CBCT sections

**Fig. 6:**
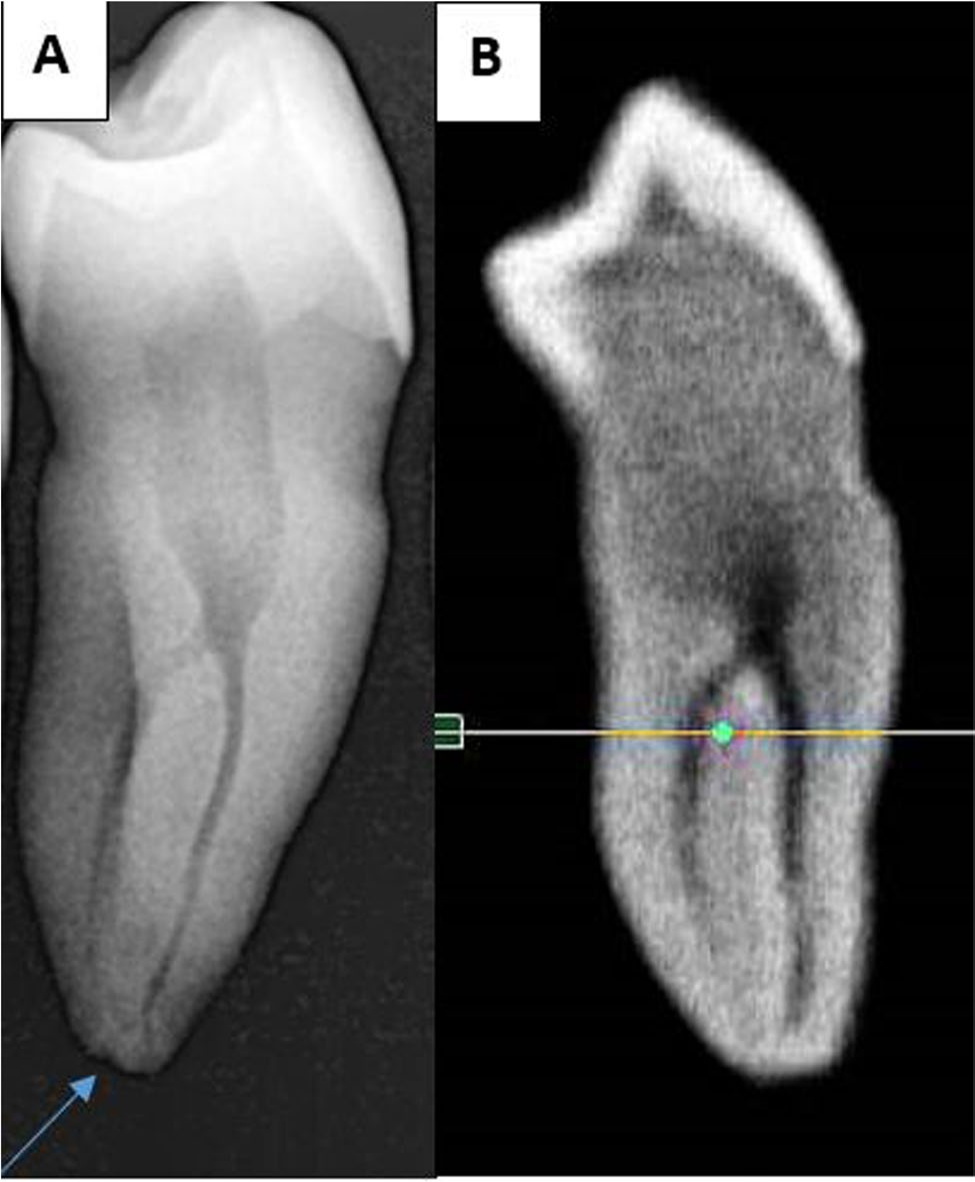
Another Vertucci class VII or Ahmed ^1^TN^1–2−1–2^. **A**: captured using digital sensor imaging **B**: one of the CBCT slices. The arrow highlights the small additional canal, which altered the classification and was detected in the digital sensor image but was not identified in the CBCT sections

It was observed that the digital sensor images were more accurate in detecting fine canals in the apical third compared to the corresponding CBCT sections (as illustrated in the attached figures (Figs. 4 and 5, and 6)). Therefore, thoroughly evaluating each case using multiple available diagnostic tools may be favourable.

**Fig. 7:**
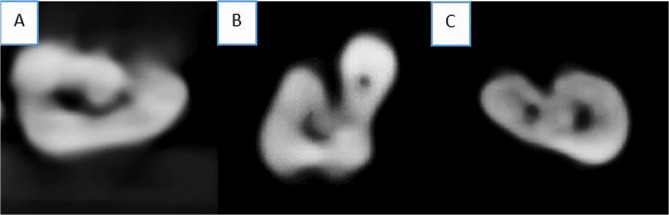
some of C-shapes canal observed in the current sample; **A**: C1 according to Fan classification, **B**: C2 according to Fan classification, and **C**: C3 according to Fan classification

Table 5 presents the prevalence of C-shaped mandibular premolars as detected in CBCT axial view images. The occurrence was low overall in the first and second MPs (4.31% and 9.82%, respectively). No significant differences were observed in the frequency of C-shaped canal types between males and females in the first or second MPs (*p* = 0.974 and *p* = 0.969, respectively), and only the first three types of Fan’s classification were observed (Fig. 7).

**Table 5:** Distribution of the first and second MPs by fan’s classification between males and females in the sample and the results of the Chi-square test

Premolar			First Premolar			Second Premolar		
Gender			Male	Female	Total	Male	Female	Total
C–shape classification according to Fan et al.	C1		2	3	5	4	5	9
	C2		1	2	3	2	3	5
	C3		1	2	3	1	1	2
Total			11 (4.31%)			16 (9.82%)		
Chi-Square Test Result		Chi-Square Value	0.052			0.0621		
		*p*-Value	0.974			0.969		

## Discussion

First MPs are particularly challenging to treat endodontically due to their diverse root canal configurations and the limited accessibility of a second canal [44]. Therefore, this study is among the first to assess the number of roots and canal morphology of MPs in the population of Damascus. Moreover, this study also conducted assessments of the sensitivity, accuracy, specificity, and overall agreement of digital sensor radiographs in comparison with the gold standard CBCT. The results of the current study suggest that the MPs can have more than a single canal. Moreover, this study highlights that two-dimensional radiographs may not be sufficient in clinical practice when assessing MP morphology because, in the clinical situation, it is not applicable to obtain a two-dimensional image in the mesial or distal view of the premolar to provide a complementary three-dimensional perspective to the image taken perpendicular to the buccal surface. While the use of three-dimensional imaging in clinical settings is generally more accurate and clinically beneficial, it should be noted that the digital sensor images appeared to be more accurate particularly in detecting fine canal branches in the apical third. As demonstrated by the results, these accessory canals—often more clearly visible on digital sensor images—could lead to changes in canal classification when compared with CBCT interpretations in certain samples. Notably, Pires et al. [45], who found that micro-CT was more sensitive than CBCT in detecting root canal anatomy. Therefore, it is clinically recommended to obtain digital sensor radiographs with different angulations as a complementary tool to three-dimensional imaging, especially in cases where the available 3D images are not sufficiently detailed. On the other hand, digital sensor radiographs acquired using the parallel technique with in the buccolingual and mesiodistal directions can be reliably used for accurately assessing the root canal morphology of extracted premolars prepared for in-vitro studies, especially as this method is readily available, easy to use, and accessible in clinical settings.

The results of the current study confirmed the primary null hypothesis regarding the lack of gender influence on the roots’ number and canal morphology of the first and second MPs in Damascus adolescents. However, the null hypothesis concerning the MPs’ length was rejected, as males exhibited significantly longer first and second mandibular premolars in the current sample.

This study also confirmed the secondary null hypothesis, demonstrating that the sensitivity, specificity, accuracy, and overall agreement between the two radiographic assessment techniques of both MPs were highly comparable. However, it is noteworthy that digital sensors performed better in detecting small accessory canals in the apical third.

The present study, which focuses exclusively on Damascus adolescents, limits the ability to assess variations in canal morphology and roots number across different ethnic and age groups. Nevertheless, the results can be compared with those from other ethnic populations, considering the evaluation methods employed. It is worth noting that this study employed two classification systems to assess the MPs morphology. The first was Vertucci’s classification [39], which was traditionally used to allow comparison the current findings with previous studies. The second was the classification proposed by Ahmed et al. [40], which offers a modern, simplified, and user-friendly coding system [46]. Although only a limited number of studies have utilized Ahmed’s system to evaluate MPs morphology [26, 29], the current study may serve as a comparative reference for future research across different populations, age groups, and ethnicities. Tables 6 and 7 presents a selection of recent studies that have examined the root canal morphology of first and second MPs across various ethnic groups based on Vertucci’s and Ahmed’s classification respectively.

**Table 6:** Description of recent studies that reported root number and Canal morphology based on vertucci’s classification, including study methodology and sample size

Study	Population	Tooth	Sample size	Assessment method	Roots number				Root canal morphology according to Vertucci’s classification								
					1 %	2 %	3 %	4 %	I %	II %	III %	IV %	V %	VI %	VII %	VIII %	Other %
Reda et al. [23]	Italian	First MP	380	CBCT – Patients	-	-	-	-	73.95%	3.95%	1.05%	16.05%	3.95%	0%	0%	0.53%	0.53%
		Second MP	340		-	-	-	-	94.12%	1.18%	0.59%	2.65%	1.18%	0%	0%	0%	0.29%
Hasheminia et al. [28]	Iranian	First MP	389	CBCT – Patients	88.69%	10.54%	0.77%	0%	81.49%	6.17%	1.02%	0.51%	8.74%	0.79%	0.51%	0%	0.77%
		Second MP	384		88.80%	9.11%	2.09%	0%	83.6%	4.42%	0.79%	0.26%	7.8%	1.04%	0%	0%	2.09%
Al-Zubaidi et al. [25]	Saudi	First MP	507	CBCT – Patients	95.5%	4.5%	0%	0%	70.00%	14.2%	2.2%	10.1%	2.8%	0%	0.4%	0.4%	-
		Second MP	493		99.2%	0.2%	0%	0%	91.10%	5.7%	0.2%	2.8%	0%	0.2%	0%	0%	-
Burklein et al. [27]	German	First MP	1044	CBCT – Patients	91.4%	8.6%	0%	0%	21.9%	5.3%	0.2%	14.7%	55.7%	2.6%	0.4%	0.2%	-
		Second MP	871		98.6%	1.3%	0.1%	0%	39.0%	1.1%	0.1%	1.4%	57.1%	0.5%	0.3%	0.3%	
Buchanan et al. [29]	Black South African	First MP	386	CBCT – Patients	97.92%	1.82%	0.25%	0%	48.5%	2.1%	9.2%	3.1%	28.0%	0.3%	0.5%	0.3%	8%
		Second MP	386		96.37%	2.33%	1.29%	0%	81.3%	1.0%	6.1%	0.5%	3.1%	0.3%	0.5%	1.0%	6.2%
Ok et al. [30]	Turkish	First MP	1471	CBCT – Patients	93.5%	6.5%	0.06%	0%	92.8%	0.3%	1%	1.4%	4.4%	0%	0%	0.06%	-
		Second MP	1345		98.5%	1.3%	0.2%	0%	98.5%	0.07%	0.07%	0.6%	0.5%	0%	0%	0.2%	-
Habib et al. [7]	North Syria	First MP	95	Injection and clearing – Extracted teeth	100%	0%	0%	0%	82.10%	1.05%	2.10%	2.10%	11.57%	1.05%	0%	0%	-
		Second MP	65		96.93%	3.07%	0%	0%	83.07%	10.76%	0%	4.62%	0%	0%	0%	1.53%	-
Llena et al. [31]	Spanish	First MP	73	CBCT – Patients	78%	20.5%	1.5%	0%	78.1%	8.2%	0%	0%	12.3%	1.3%	0%	0%	1.3%
		Second MP	53		90.5%	9.5%	0%	0%	90.6%	1.8%	0%	0%	7.5%	0%	0%	0%	0%
Thanaruengrong et al. [32]	Thai	First MP	621	CBCT – Patients	98.1%	1.6%	0.3%	0%	79.3%	0.9%	1.6%	0.3%	16%	0.2%	0.1%	0%	1.7%
		Second MP	538		98.9%	0.9%	0.2%	0%	63.1%	1.4%	2.6%	0.6%	28.5%	0.3%	0.2%	0%	3.2%
Alenezi et al. [33]	Kuwaiti Subpopulation	First MP	245	CBCT – Patients	73.88%	23.31%	0.78%	0.39%	14.1%	18.7%	14.1%	8.4%	5.9%	14.3%	1.9%	0%	22.6%
		Second MP	231		80.78%	19.22%	0%	0%									
Gawdat et al. [47]	Egyptian	First MP	306	CBCT – Patients	95.4%	4.6%	0%	0%	62.4%	0.7%	9.2%	0%	27.1%	0.3%	0.3%	0%	-
		Second MP	288		98.6%	1.4%	0%	0%	96.2%	0%	1.7%	0%	2.1%	0%	0%	0%	-

*MP* Mandibular Premolar

**Table 7:** Description of recent studies that reported root number and Canal morphology based on ahmed’s classification, including study methodology and sample size

Study	Population	Tooth	Sample size	Assessment method	Root canal morphology according to Ahmed’s classification																
					^1^TN^1^ %	^1^TN^1–2^ %	^1^TN^1–2−1^ %	^1^TN^1–2−1–2^ %	^1^TN^1–3^%	^1^TN^2^%	^1^TN^2−1^%	^1^TN^2−1−2^%	^2^TNB^1^L^1^ %	^2^TN^1^B^1^L^1^ %	^2^TNB^1–2^L^1^ %	^2^TNB^2^L^1^ %	^2^TNM^1^D^1–2−1–2^ %	^2^TNM^2^/D^2^ %	^2^TNM^2^D^2^ %	^3^TN MB//DB^1–2−1^L^1^ %	^3^TN MB^1^DB^1^L^1^ %
Karobari et al. [26]	Saudi subpopulation	First MP	645	CBCT – Patients	81.5%	5.3%	5.3%%	0.47%	0%	0%	0%	0%	0.93%	6.5%	0%	0%	0%	0%	0%	0%	0%
		Second MP	585		95.9%	0.85%	0.68%	0%	0%	0%	0%	0%	0.34%	0.34%	0%	0%	0%	0%	0%	0%	1%
Buchanan et al. [29]	Black South African	First MP	386	CBCT – Patients	48.5%	9.6%	9.1%	0.5%	0.25%	0.5%	1%	0.25%	0%	1.6%	0.25%	0%	0%	0%	0%	0%	0.25%
		Second MP	386		81.3%	1.8%	5.6%%	0.5%%	0%	0.25%	1%	0.25%	0%	1%	0%	0.25%	0.25%	0.5%	0.25%	0.25%	1%

Notably, the current study aligns with many recent published studies in finding that most MPs have one root, the most common Vertucci classification in MPs is Type I, the second premolar tends to have less anatomically complex than the first premolar. The presence of three roots in MPs is rare but can occur. There is, however, conflicting data on whether Vertucci’s Type V or Type IV is the second most common classification after Type I in these teeth. This variation may be attributed to ethnic differences, the diverse origins of the populations studied [48], and the adopted assessment methods mentioned in the previous table.

According to studies by Ok et al. [30] and Bürklein et al. [27], men exhibited slightly more roots and root canals in mandibular first premolars compared to women. Similarly, Martins et al. [49] reported that women had fewer roots and a higher prevalence of Type I canal configurations, while men presented with three different types of root canal systems, though no significant gender differences were noted. In contrast, Al-Zubaidi et al. [25] found no correlation between gender and the number of roots or canal morphology in first and second premolars, aligning with the findings of the present study.

This study found that men tend to have longer MPs than women, which aligns with the findings of Awawdeh et al. [4] in the Jordanian population and Llena et al. [31] in the Spanish population. However, it contrasts with earlier research conducted on the Caucasian population [50]. These differences may be attributed to ethnic variations across populations.

Notably, second MPs exhibited a higher occurrence of C-shaped configurations compared to first MPs. Although the overall prevalence was low (4.31% and 9.82%, respectively), it exceeded the rates reported in studies conducted on Arab populations investigating the presence of C-shaped canals in MPs. For example, among Kuwaitis [33], C-shaped canals were found in 1.3% of second MPs and were entirely absent in first MPs. Similarly, in an Egyptian population [47] and Saudi population [26], C-shaped canals were only observed in second MPs (0.3% and 0.4%, respectively), with no cases reported in the first MPs. In contrast, studies from other countries have shown different patterns. Among the Thai [32] population, for example, C-shaped canals were found in 23.7% of first MPs and only 0.7% of second MPs. This discrepancy may suggest a potential ethnic influence on the distribution of C-shaped configurations in MPs. However, the findings of the present study are consistent with the mentioned previous studies in showing that gender has no significant influence on the presence of C-shaped canals.

One limitation of the current study is that it exclusively investigated extracted MPs within a specific age group from the population of Damascus, who were undergoing extraction of MPs as part of orthodontic treatment, which may not fully reflect the anatomical variations present in the general population. Additionally, the targeted sample in this study may not allow for the inclusion of a larger sample size. To address this limitation, future multi-center retrospective studies using high-quality three-dimensional images of full-arch CBCT scans from dental center archives along with digital sensor imaging at multiple angulations could be conducted with larger sample sizes across several Syrian provinces and different age groups to more comprehensively evaluate the anatomical shape of MPs in the Syrian population. Comparing these results with the current study could provide a more comprehensive understanding of diagnostic accuracy across different clinical contexts.

## Conclusion

Considering the limitations of this study, which focused on extracted mandibular premolars from adolescents in Damascus, most first and second mandibular premolars exhibited a single root with a Type I (^1^TN^1^) canal configuration, followed by Type V (^1^TN ^1–2^) and Type IV (^1^TN^2^) canal configurations. This distribution is somewhat similar to that reported in previous studies from neighbouring countries and regions, as indicated by published data. Although gender did not influence the number of roots or canal morphology, it was associated with premolar length, with males exhibiting longer roots.

When preparing the extracted mandibular premolars for an in-vitro study, digital periapical radiography is recommended for accurate evaluation of root canal anatomy, particularly for detecting small accessory canals in the apical third of the root. Clinically, digital dental radiography taken from multiple angulations is also recommended as a complementary tool to CBCT imaging, enabling a more precise diagnosis of root canal morphology.

## Data Availability

De-identified data are available upon reasonable request to the corresponding author.
